# Essential surgery delivery in the Northern Kivu Province of the Democratic Republic of the Congo

**DOI:** 10.1186/s12893-024-02386-3

**Published:** 2024-03-22

**Authors:** Luc Kalisya Malemo, Ava Yap, Boniface Mitume, Christian Salmon, Kambale Karafuli, Dan Poenaru, Rosebella Onyango

**Affiliations:** 1 School of Medicine, The University of Goma, Goma, Democratic Republic of Congo; 2 Center of Health Equity in Surgery and Anesthesia, University of California San Francisco, San Francisco, USA; 3 Department of Computer Engineering, Université Officielle de Ruwenzori, Butembo, Democratic Republic of Congo; 4 Centre for Global Health Engineering, Department of Engineering Management and Industrial Engineering, Western New England University, Springfield, MA USA; 5 Université Libre des Pays des Grands Lacs, Goma, Democratic Republic of Congo; 6 Department of Pediatric Surgery, McGill University, Montreal, QC Canada; 7 Department of Community Health and Development, Great Lakes University of Kisumu, Kisumu, Kenya

**Keywords:** Global surgery, Hospital capacity, Democratic Republic of the Congo, Access, Surgical disease burden

## Abstract

**Introduction:**

Surgical services are an essential part of a functional healthcare system, but the Lancet Commission of Global Surgery (LCoGS) indicators of surgical capacity such as perioperative workforce and surgical volume are unknown in many low- and middle-income countries (LMICs) including the Democratic Republic of Congo (DRC). We aimed to determine the surgical capacity and its associated factors within the DRC.

**Methods:**

Hospitals were assessed in the North Kivu province of the DRC. Hospital characteristics and surgical rates were determined using the WHO-PGSSC hospital assessment tool and operating room (OR) registries. The primary outcome of interest was the number of Bellwether operations (i.e. Caesarean sections, laparotomies, and external fixation for bone fractures) per 100,000 people. Univariate and multiple linear regressions were performed. Primary predictors were the number of trained surgeons, anaesthesiologists, and obstetricians (SAOs) and the number of perioperative providers (including clinical officers and nurse anaesthetists) per 100,000 people.

**Results:**

Twenty-eight hospitals in North Kivu were assessed over one year in 2021; 24 (86%) were first-level referral health centres while 4 (14%) were second-level referral hospitals. In total, 11,176 Bellwether procedures were performed in the region in one year. Rates per 100,000 people were 1,461 Bellwether surgical interventions, 1.05 SAOs, and 13.1 perioperative providers. In univariate linear regression analysis, each additional SAO added 239 additional cases annually (*p* = 0.023), while each additional perioperative provider added 110 cases annually (*p* < 0.001). In our multiple regression analysis adjusting for other hospital services, the association between workforce and Bellwether surgeries was no longer significant.

**Conclusions:**

The surgical workforce in DRC did not meet the LCoGS benchmark of 20 SAOs per 100,000 people but was not an independent predictor of surgical capacity. Major investment is needed to simultaneously bolster healthcare facilities and increase surgical workforce training.

## Introduction

Through the Lancet Commission on Global Surgery (LCoGS) and the World Health Organization (WHO) resolution 68.15, essential surgical care was unanimously endorsed as a component of universal health coverage [1, 2]. The LCoGS targets that by 2030, 80% of surgical needs should be met, focusing on improving access to essential surgery. Despite this recognition of need, five billion people continue to lack access to essential surgery, 29% of them living in Sub-Saharan Africa [3, 4].

While many low- and middle-income countries (LMICs) are now trying to incorporate essential surgery in their national strategic plans, evidence-based surgical plans are hindered by the absence of accurate estimates of untreated surgically correctable conditions, preventing sound investments to strengthen health systems. A recent systematic review of African National Health Strategic Plans (NHSPs) revealed that 19% of these NHSPs did not mention surgery at all, and 65% mentioned it five times or less [5]. Out of the total 4,064 health targets surveyed, only 2% were related to surgical conditions or surgical care [5]. This is also the case for the Democratic Republic of the Congo (DRC), where no informed surgical targets exist in the National Health Plan [6].

The volume and short-term outcomes of essential surgery and the quality of perioperative care in the DRC remains unknown. Similarly, there is little information regarding current surgical infrastructure, functional equipment, surgical and anaesthetic workforce, training structures, referral systems and financing mechanisms [7, 8]. Without this information, it is difficult to set priorities for the nation’s surgical care and convince stakeholders to invest in surgery.

To make assessment more efficient, three essential surgical procedures, named Bellwether procedures (Caesarean section, laparotomy, and external fixation of open fractures) have been considered by the WHO and international surgical experts as proxies for assessing a facility’s capacity to meet the surgical needs of its populations [3]. The LCoGS core indicator of timely essential surgery access also targets a minimum of 80% of the population that can reach a facility with Bellwether procedures within 2 hours [2]. Therefore, we used these three procedures to estimate the proportion of the community’s surgical needs that was actually met by hospitals in the North Kivu province of the DRC. This study evaluated the association between the number of Bellwether surgeries with the hospital-based surgical capacity in North Kivu with a focus on assessing the facilities’ surgical workforce, infrastructure, and equipment.

## Materials and methods

### Study design, setting, and participants

This study evaluates the current surgical capacity of hospitals and the gaps in the delivery of surgeries at hospital in two health zones in the North Kivu province of DRC (Katwa and Butembo). This region has a total population of 764,561, 46.4% of whom are children aged 14 years old or younger [9]. An assessment of all hospitals officially allowed to perform surgery as per the local Ministry of Health (MoH) regulations served as an indicator of the yearly met and unmet surgical needs in this region. Healthcare facilities without an in-house physician were excluded as they did not have capacity to perform Bellwether procedures. Ethical approval was obtained by the Great Lakes University of Kisumu Ethics Board at Kisumu and the North Kivu Provincial MoH Office of Research Ethics of the DRC. Verbal consent was obtained from all participants.

### Procedures and data sources

Between February to May 2022, we assessed the surgical capacity of public and non-public health centres using the Program in Global Surgery and Social Change (PGSSC-WHO) Hospital Assessment tool [10]. The annual case volumes from January to December 2021 were manually extracted and recorded electronically. Medical students were trained as research assistants to collect the necessary data from individual sites. Data sources included patient data from operating room (OR), admissions, out-patient and discharge registries, and relevant datasets or verbal accounts in absence of any written documentation provided by personnel from financial, human resources, social work, and perioperative departments, including surgeons, anaesthetists, OR nurses, ward nurses, and other hospital staff. Specifically, case volumes were derived from operating room logbooks and inpatient ward registries. Variables collected included the number of perioperative providers, demographics, patient addresses, and clinical details including diagnosis, surgical indication, surgical procedure, anaesthesia type, postoperative mortality during the index hospitalization, and whether the surgery was performed by a surgeon or a non-surgeon. Some OR registries also provided details on the financial coverage and costs of surgery.

The primary outcome of interest was the number of Bellwether procedures performed and the cumulative number of Bellwether procedures per 100,000 people. The primary predictors of this outcome were the number of SAOs and other perioperative providers, defined as any medical provider who contributed to surgery, including nurse anaesthetists, clinical officers, and non-surgeon physicians. These predictors were also reported in both absolute numbers and density per 100,000 people.

### Data analysis

Data were analysed using Stata version 16.1 (College Station, TX). Individual in-hospital services availability was aggregated into service categories by assigning an average percentage to each type of service (e.g. radiology was a composite availability percentage of plain film radiography, computed tomography, and magnetic resonance imaging). Hospitals were categorised by ownership status, and comparisons between these groups were conducted with nonparametric tests such as Fisher’s exact test and Wilcoxon Rank-Sum test. A *p*-value of < 0.05 was considered significant. Costs were reported in United States dollars ($).

The primary outcome (i.e. the number of Bellwether surgeries performed) was described. Linear regression analyses were then performed to determine hospital-specific characteristics that were associated with Bellwether surgical volume. For the multiple regression analysis, covariates were selected based on their individual significance in association with the primary outcome in univariate analysis, such that any predictor that showcased an association that was *p* < 0.05 with the primary outcome was included into the multivariable model. Complete case analysis was used following listwise deletion, such that observations with a missing value in one or more model variables were excluded.

## Results

Twenty-eight hospital facilities were assessed across the two health zones, 15 (54%) located in Butembo and 13 (46%) in Katwa. Most hospitals were referral health centres (*n* = 24, 86%), which are first-level healthcare points receiving patients before the referral hospitals and are predominantly staffed by medical officers performing surgery without formal surgical training. Formal surgical training is defined as completing a surgical training program that is accredited by a national or regional medical board or surgical association. The remaining four hospitals (14%) were second-level referral hospitals staffed with both more general physicians and physicians who had completed training programs. The public sector did not own any referral hospitals (Table 1).

**Table 1:** Baseline characteristics of hospitals in the North Kivu province of the Democratic Republic of the Congo, subdivided by district. For continuous variables the median and interquartile range (in parentheses) are reported. For categorical variables, the number and percentage proportion (in parentheses) are reported. The two groups were compared using Wilcoxon rank-sum tests for continuous variables and Fisher’s exact tests for categorical variables.

	Butembo (*N* = 15)	Katwa (*N* = 13)	Total (*N* = 28)	*p*-value
Ownership				0.26
Public	4 (27%)	0 ( 0%)	4 (14%)	
Faith-based	6 (40%)	7 (54%)	13 (46%)	
Non-profit	1 ( 7%)	2 (15%)	3 (11%)	
Private	4 (27%)	4 (31%)	8 (29%)	
Level				1.00
Health Centre	13 (87%)	11 (85%)	24 (86%)	
Referral Hospital	2 (13%)	2 (15%)	4 (14%)	
% Patients Insured at Each Site (*n* = 27)	0 (0–13)	13 (13–13)	13 (0–13)	0.029
Cost of Caesarean section (USD) (*n* = 27)	78 (65–80)	50 (35–60)	60 (35–80)	0.036
Cost of external fixation (USD) (*n* = 5)	80 (80–100)	100 (100–100)	100 (80–100)	0.18
Cost of laparotomy (USD) (*n* = 28)	80 (60–100)	95 (65–100)	90 (63–100)	0.44
Available surgical beds (#) (*n* = 28)	22 (18–57)	37 (23–65)	27 (20–59)	0.30
Available PACU beds (#) (*n* = 28)	2 (2–3)	2 (1–)	2 (1–3)	0.32
Available ICU beds (#) (*n* = 28)	0 (0–3)	2 (1–2)	1 (0–3)	0.24
Available ORs (#) (*n* = 28)	1 (1–2)	1 (1–2)	1 (1–2)	0.69
% Utilities availability (*n* = 28)	50 (25–72)	53 (47–69)	52 (31–70)	0.38
% Perioperative meds availability (*n* = 28)	75 (64–84)	63 (43–70)	66 (52–80)	0.018
% Imaging services availability (*n* = 28)	0 (0–0)	0 (0–0)	0 (0–0)	0.85
% Lab and transfusion availability (*n* = 28)	3 (0–3)	3 (0–13)	3 (0–10)	0.37
% Functional airway equipment (*n* = 28)	29 (18–50)	13 (10–50)	20 (12–50)	0.33
% Functional anaesthesia equipment (*n* = 28)	40 (33–60)	40 (35–55)	40 (34–59)	0.91
% Functional surgical equipment (*n* = 28)	79 (77–83)	81 (77–84)	80 (77–83)	0.38
% Functional basic med. equipment (*n* = 28)	83 (67–100)	94 (88–100)	94 (83–100)	0.38
% 24 h periop. services availability (*n* = 28)	33 (29–38)	21 (4–33)	33 (13–33)	0.054
No. of surgeons (*n* = 28)	0 (0–0)	0 (0–0)	0 (0–0)	0.88
No. of anaesthetists (*n* = 28)	0 (0–0)	0 (0–0)	0 (0–0)	
No. of obstetricians (*n* = 28)	0 (0–0)	0 (0–0)	0 (0–0)	0.34
No. of non-surgeon providers (*n* = 28)	2 (1–3)	1 (1–3)	1 (1–3)	0.35
No. of nurse anaesthetists (*n* = 28)	1 (0–1)	1 (1–2)	1 (0–2)	0.19
No. of SAO providers (*n* = 28)	0 (0–0)	0 (0–0)	0 (0–0)	0.40
No. of perioperative providers (*n* = 28)	3 (2–4)	2 (2–5)	3 (2–5)	0.94
No. of laparotomies (*n* = 28)	44 (17–104)	105 (35–274)	63 (29–176)	0.11
No. of Caesarean sections (*n* = 28)	130 (78–211)	149 (17–418)	137 (69–295)	0.60
No. of external fixations (*n* = 28)	0 (0–0)	0 (0–0)	0 (0–0)	0.88
No. of Bellwether procedures (*n* = 28)	211 (98–343)	372 (60–609)	230 (97–601)	0.66
Inpatient post-operative deaths (#) (*n* = 28)	0 (0–0)	0 (0–0)	0 (0–0)	0.65

USD = United States dollars, WHO = World Health Organization, M&M = morbidity & mortality, PACU = post-anaesthesia care unit, ICU = intensive care unit, OR = operating room, SAO = surgeons, anaesthetists, and obstetricians

*There were no anaesthetists available at any hospitals, which is why there is no *p*-value reported for this variable

Hospitals had a median of 27 surgical floor beds per facility (or a cumulative 6.0 surgical beds per 1000 people). The hospitals also had a median of 2 post-anaesthesia care unit (PACU) beds, 1 intensive care unit (ICU) beds, and 1 ORs (or a cumulative 1.7 ORs per 100,000 people). Imaging services were unavailable for most hospitals. Lab and transfusion availability was also low (median of 3%). Anaesthetic equipment was available 40% of the time, while surgical equipment was available 80% of the time. The formal surgical workforce within the two health zones consisted of three trained surgeons, five gynaecologists-obstetricians and no physician-anaesthesiologists, which translated to a SAO density of 1.05 per 100,000 people. Non-surgically trained medical doctors performed all the Bellwether procedures at all the public hospitals as no specialised surgical provider was present. Another 59 medical doctors performed surgery and 33 nurse anaesthetists provided anaesthesia. When accounting for both trained and untrained specialists, the workforce density was 13.1 perioperative providers per 100,000 people.

Across the two healthcare zones, 11,167 Bellwether procedures were cumulatively performed over a year, comprising 6,105 Caesarean sections, 4,536 laparotomies, and 526 external fixations. Annually, 1,461 adult Bellwether surgeries per 100,000 people were performed. The number of operations per 100,000 people for Caesarean sections, laparotomies, and external fixations of open bone fractures was 798, 593, and 69, respectively. The inpatient post-operative mortality was 1.0 per 100,000 people.

During the study period, a median of 13% of patients had health insurance to cover the cost of their surgery. The median costs for Bellwether surgeries in the region were $60 for a Caesarean section, $90 for a laparotomy and $100 for external fixation of a bone fracture. The procedural costs were not significantly different between the public and non-public sectors. Of note, none of the patients accessing surgical care in public hospitals had health insurance, which meant that they paid fully out of pocket (OOP).

The primary predictor was selected as the number of perioperative providers because this variable was a LCoGS indicator and demonstrated significant associations with the primary outcome of Bellwether surgical volume. Univariate linear regression modelled the association of Bellwether surgical volume with the number of perioperative providers and SAOs. Each additional perioperative provider was associated with 110 additional Bellwether cases annually (*p* < 0.001, 95% confidence interval [CI] 55–165). (Table 2) All individual Bellwether procedures had a statistically significant association with increases in the perioperative workforce (Table 3). Each additional SAO in the workforce was associated with 239 additional Bellwether cases annually (*p* = 0.023, 95% CI 35–441). When regression models were applied to other hospital resources, Bellwether surgeries were positively significantly associated with imaging availability (*p* = 0.001), lab availability (*p* < 0.001), number of surgical beds (*p* < 0.001), anaesthetic equipment (*p* = 0.009), and surgical equipment (*p* = 0.004), but not significantly associated with medication availability (*p* = 0.645) or number or ORs (*p* = 0.071). (Table 2)

**Table 2:** Changes in the number of Bellwether surgeries in the DRC associated with additional perioperative services based on univariate linear regression modelling.

Perioperative service	Change in number of cases	95% confidence interval	*p*-value
Additional SAO provider	239	36–441	0.023
Additional perioperative provider	110	55–165	< 0.001
Additional operating room	157	-15–330	0.071
Additional surgical bed	7.3	4.8–9.9	< 0.001
Medications*	234	-796–1263	0.645
Laboratory & blood bank*	1167	559–1745	< 0.001
Diagnostic imaging*	824	396–1252	0.001
Anaesthesia equipment*	1174	320–2029	0.009
Surgical equipment*	3487	1235–5739	0.004

*From 0 to 100% availability

**Table 3:** Changes in number of surgical interventions associated with an SAO provider or perioperative provider added to the workforce for each of the Bellwether surgeries (laparotomy, Caesarean section, and external fixation) based on univariate linear regression models.

Bellwether Surgery and change in the workforce	Change in number of cases	95% confidence interval	*p*-value
Laparotomy			
An additional SAO provider	93	-20–206	0.102
An additional perioperative provider	41	8–74	0.018
Caesarean section			
An additional SAO provider	122	22–221	0.019
An additional perioperative provider	60	35–85	< 0.001
External fixation			
An additional SAO provider	24	-1–49	0.059
An additional perioperative provider	9	1–16	0.031

After controlling for hospital level, health zone, ownership status, number of surgical beds, imaging, laboratory services, blood bank, and surgical and anaesthetic equipment availability in a multiple linear regression model, the number of perioperative providers was no longer associated with the number of Bellwether surgeries performed (beta-coefficient 28.75, *p* = 0.650, 95% CI -106-164) (Table 4). Conversely, the association with the number of surgical beds neared but did not cross the level of statistical significance (beta-coefficient 7.59, *p* = 0.053, 95% CI -0.109–15.28). The goodness of fit for this multiple regression model was determined by the R² or the coefficient of determination, which was 0.734.

**Table 4:** Multiple linear regression model showcasing variables associated with changes in the number of Bellwether surgeries performed in the North Kivu region of the DRC (R² = 0.734).

Variables in multiple linear regression model	β coefficient (No. of Bellwether surgeries)	*p*-value	95% confidence interval
No. of perioperative providers	28.75	0.650	-106.0–163.5
Referral hospital (Health centre as reference)	2,108	0.675	-8,571 − 12,787
Katwa district (Butembo as reference)	263.0	0.238	-198.7–724.7
Faith-based hospital ownership*	-173.8	0.558	-801.6–453.9
Non-profit hospital ownership*	-83.36	0.821	-870.5–703.8
Private hospital ownership*	139.0	0.638	-488.4–766.3
% Functional surgical equipment	26.96	0.150	-11.25–65.16
% Functional anesthesia equipment	8.996	0.330	-10.32–28.31
Available Operating rooms (#)	-88.89	0.423	-322.3–144.5
Available surgical beds (#)	7.584	0.053	-0.109–15.28
% Utilities availability	-4.446	0.419	-16.03–7.134
% Imaging services availability	-33.66	0.704	-222.3–154.9
% Lab and transfusion availability	-10.98	0.576	-52.58–30.62
% Perioperative meds availability	5.740	0.443	-10.04–21.52

*Public hospital ownership was used as reference

## Discussion

The study assessed the relationship between the number of Bellwether surgeries, the surgical capacity, inhuman capital, and perioperative services in two Congolese health zones in 2021 and is one of the first to evaluate the surgical workforce, infrastructure, and equipment in the DRC. On average, 1,461 Bellwether surgeries per 100,000 people were performed annually, which was within the range of other studies in Sub-Saharan Africa reporting adult surgical volumes of 43 − 4,268 per 100,000 [11–13]. This supports a previous finding that greater than 95% of the population in Central Sub-Saharan Africa does not have access to surgical care [4]. While our case count does not capture all adult surgeries, Bellwether surgeries have been shown to comprise approximately half of the total adult surgeries and can serve as a proxy of overall surgical volume [14]. Considering this adjustment, the projected adult surgical volume in the North Kivu region remains well below the LCoGS recommended rate of at least 5,000 essential surgeries per 100,000 people per year [15]. Reasons for inadequate surgical access are manifold, as previous studies in North Kivu have identified distance to hospitals, poverty, and gender disparity as key barriers to accessing surgery [16]. In particular, rural patients have reported an inability to seek medical care because of the long distance to the nearest hospital and lack of transportation [17].

The number of SAO provider density in our study (1.05 per 100,000 people) was also well below the Lancet recommendations of 20 SAO per 100,000 population and on par with the regional SAO density reported in current literature. In comparison, the average African surgical workforce ratio was 0.7 SAOs per 100,000 people [18–20]. Relatedly, a systematic review of SAOs practising in LMICs found a surgeon density of 0.13 to 1.57 per 100,000 people and an obstetrician density of 0.18 per 100 000 population [8]. Notably, the surgical provider density rose to 13/100,000 when accounting for all providers performing surgery including those who are not formally trained. This metric was closer to but still did not meet the Lancet recommendations. The slim national budget may explain the scarcity of certified surgical providers in the DRC, where only 1% of the MoH budget was allocated to healthcare in 2014 [21]. This percentage is significantly lower than other African governments that have committed to spending 15% of their budget on health [22, 23].

Notably, each additional surgical provider and SAO provider were associated with an increase of 110 cases and 239 cases, respectively. This supports that growing the surgical workforce can help expand the surgical volume and provide stakeholders with a benchmark on the expected case volume increase to meet the LCoGS indicators. This positive association disappeared in multiple regression analysis including other hospital variables, which likely acted as mediators to the surgical outcome. As surgeons will require other coordinated hospital supplies and infrastructure to perform surgery, these perioperative services would need to be simultaneously bolstered to confer a significant increase in surgical volume. Indeed, the borderline association between number of Bellwether procedures and number of surgical beds alludes to the possibility that surgical infrastructure and equipment may be just as, if not more important.

Perioperative supplies and equipment were also inadequately stocked in the DRC hospitals. Items on the WHO list of essential resources needed for high-quality surgical care were far from universally available, ranging from 8% for imaging services to 86% for basic medical equipment, and were less available than in similar countries such as Uganda and Ghana [24, 25]. DRC hospitals also had only 1.7 surgical beds per 1,000 people, which was comparable to hospitals in neighbouring countries of Tanzania, Uganda and Mozambique [26] and 4 to 10 times less than the bed ratio per 1,000 people reported in high-income countries [27]. Study hospitals also collectively had 6.0 ORs per 100,000 people, which was higher than a previous study in LMICs that reported < 1 OR per 100,000 people but lower than > 14 ORs per 100,000 people in high-income countries [24]. Again, the limited infrastructure could be explained by the low budget allocated to healthcare in the DRC [21]. In fact, a previous study demonstrated that the greatest surgical disparities in Eastern DRC were due to infrastructure deficiencies [28].

A large majority of the hospitals were in the non-public sector (82%), which comprises hospitals run by churches and other non-public charity organizations. Substantial charity support of LMIC surgery is well documented, as a previous systematic review of the faith-based organizations showed a wide variability of their contributions: 30% or less in Sudan, Botswana, Sierra Leone, Central African Republic, Togo, Chad, 40% or more in Kenya, Tanzania, Malawi, Nigeria, Rwanda, Benin, 50% and more in the DRC and in Uganda [29]. In fact, the province of North Kivu is a permanent home for hundreds of NGOs, which leaves the healthcare system reliant on these organizations [29]. Nationally, the DRC ranks as the sixth failed state in the world because of its inability to provide public services [18, 26].

Only 11% of clients had health insurance in this study, while the costs of the Bellwether surgeries ranged between $61-$92. None of the patients in public hospitals had insurance and therefore needed to pay for surgery OOP, putting them at risk of catastrophic healthcare expenditures [30]. OOP spending for a surgical procedure is not feasible for a large proportion of the Congolese people, whose average GDP per capita was $418 in 2022 [31]. Another study in the DRC found that four out of ten households were not able to pay the premium fee to maintain health insurance coverage [32]. Thus, the impact of surgical conditions on catastrophic healthcare expenditure needs to be quantified to help inform financial risk protection mechanisms that appropriately incorporate coverage for surgery.

The in-hospital post-operative mortality of 1.05 per 100,000 people in our study was lower than in similar studies in neighbouring countries. For example, one study conducted in Uganda reported higher figures: 2.6 deaths per 100,000 people for surgeries overall, 2.6 per 100,000 people after Caesarean sections, and 11.3 deaths per 100,000 people after laparotomies [33]. Another study in Tanzania reported post-laparotomy mortality varying between 7 deaths and 9 deaths per 100,000 population [34]. Our study’s low mortality proportion could be explained by the pre-selection of patients treated – only those who could arrive at the hospitals to seek care. Those who were relatively stable and well-resourced fare a better chance to access appropriate surgical care and survive postoperatively. In fact, a related community-based study submitted for publication reported up to 35% mortality from surgical disease, substantiating that pre-hospital deaths may be much more prevalent.

### Limitations

Our first limitation is the retrospective design of the study, which led to difficulty in documenting some hospital-based variables. For example, mortality data was only gathered during the index hospitalization that included the Bellwether surgery, omitting anyone who died after discharge or readmission, and therefore may significantly underreport the mortality incidence. Nevertheless, information was mostly derived from a permanent OR registry log that improved the quality of information recorded. Secondly, no standardised database existed to record hospital data in the two health zones. Most data were manually entered in operating registries and transcribed in Excel sheets, which is prone to errors. We minimised this bias by collecting data from a diverse array of sources and stakeholders involved in the delivery of surgery. We also verified some information by calling key responders. Thirdly, health personnel who answered questions about available and functional resources varied across hospitals, which may have contributed to some missing data observed in some of the hospitals. However, the level of missingness was nominal such that the negligible amount of the surgical records missing most likely did not affect the validity of the study. Fourthly, we did not collect pre-hospital data for this study, which limited our scope to healthcare access only once the patient has arrived at the hospital. Finally, due to limitations in the completeness of the registry sources, we were unable to reliably collect the case counts for all adult surgeries, preventing us from directly measuring the LCoGS indicator of surgical volume.

## Conclusions

This study found that delivery of surgical care in the North Kivu province of DRC falls far short of the minimum goals set forth by international agencies and the LCoGS. In many cases, the surgical capacity was less than in other countries with comparable economic profiles. It is apparent that there are huge unmet needs in both physical resources and human resources that contribute to the profound unmet surgical needs of this province. There is an urgent need to put in place plans for improving surgical services in the DRC. The infrastructure and human capital needs are largely unmet and will require a much larger commitment of resources from the national government and MoH than currently allocated. The results of this study can provide a snapshot of DRC’s surgical capacity to inform recommendations on what is needed to solve the gap based on African guidelines.

## Data Availability

Full transcript data and materials are not openly available to the public due to potential for sites to be identified by their characteristics with the possibility to openly compare post-surgical outcomes among health centers, even when overtly identifying data could be omitted. Data may be available from the corresponding author upon reasonable request.
